# Supplementary material for: Effects of Kadsura coccinea L. Fruit Extract on Growth Performance, Meat Quality, Immunity, Antioxidant, Intestinal Morphology and Flora of White-Feathered Broilers
Source: Animals (Basel). 2022 Dec 26;13(1):93. doi: 10.3390/ani13010093 (PMC9817888; doi:10.3390/ani13010093)
Supplement: Supplementary file 1 [file animals-13-00093-s001.zip › animals-2102812-supplementary.pdf]

Table S1. Effects of the fruit extract of *K. coccinea* on slaughter performance of white-feather broilers

items	CK	PD	LD	MD	HD
Dressing percentage	90.57 ± 2.36	91.29 ± 1.69	92.78 ± 1.64	93.27 ± 4.05	93.19 ± 1.37
Semi-eviscerated percentage	82.19 ± 3.34^{b}	83.94 ± 2.81^{ab}	83.65 ± 1.09^{ab}	85.68 ± 2.94^{a}	84.13 ± 1.86^{ab}
Eviscerated percentage	71.61 ± 3.62	72.16 ± 2.51	71.57 ± 1.37	74.33 ± 3.39	72.29 ± 1.39
Breast muscle percentage	22.18 ± 1.68^{a}	21.26 ± 1.20^{ab}	20.21 ± 1.78^{b}	22.12 ± 1.01^{a}	20.54 ± 0.82^{b}
Thigh muscle percentage	14.65 ± 0.91^{ab}	13.59 ± 0.78^{b}	14.69 ± 0.99^{ab}	14.94 ± 1.88^{a}	14.97 ± 0.68^{a}
Abdominal fat percentage	1.52 ± 0.46^{a}	0.73 ± 0.22^{b}	0.84 ± 0.45^{b}	0.79 ± 0.39^{b}	0.53 ± 0.17^{b}

Different letters in the same column indicate significant differences between groups according to the Tukey test. $^{a, b}$ Means in the same row with different superscript letters indicate differences ($p < 0.05$).

Table S2. Effects of the fruit extract of *K. coccinea* on leg muscle quality of white-feather broilers

items	CK	PD	LD	MD	HD
L*	52.57±5.54	49.50±6.92	49.18±2.33	51.77±1.46	50.38±2.57
a*	17.00±4.48	18.49±3.96	17.06±3.95	17.13±2.89	17.02±1.62
b*	11.22±3.18	11.27±1.78	9.63±1.95	10.79±1.68	10.21±1.70
pH reduction	0.55±0.52^{a}	0.5±0.13^{a}	0.25±0.13^{b}	0.56±0.18^{a}	0.38±0.10^{ab}
Shear force/N	25.38±3.78^{b}	42.59±14.44^{a}	36.95±8.28^{ab}	41.57±17.37^{a}	39.60±15.04^{ab}
Drip loss	11.65±6.7	13.79±4.86	18.75±6.10	14.36±10.53	14.10±12.28
Pressure loss	11.40±6.16	9.59±4.53	10.73±4.12	11.26±5.36	10.99±4.27

Different letters in the same column indicate significant differences between groups according to the Tukey test. $^{a, b}$ Means in the same row with different superscript letters indicate differences ($p < 0.05$)

Table S3. Effects of the fruit extract of *K. coccinea* on breast muscle quality of white-feather broilers.

items	CK	PD	LD	MD	HD
L*	52.06±4.87	50.15±3.67	49.56±2.73	52.86±2.39	51.47±3.33
a*	13.81±1.41	17.33±4.83	16.18±3.06	16.45±4.13	15.45±4.13
b*	9.027±1.61^{b}	11.46±2.13^{ab}	9.58±2.34^{ab}	12.15±2.68^{a}	10.58±1.88^{ab}
pH reduction	0.49±0.42^{b}	0.60±0.37^{ab}	0.79±0.14^{ab}	0.90±0.13^{a}	0.66±0.34^{ab}
Shear force/N	25.44±9.94	30.22±14.22	30.61±8.09	34.08±7.45	31.07±9.19
Drip loss	9.00±3.93	14.29±6.33	15.32±5.02	11.11±6.97	15.33±14.72
Pressure loss	9.86±4.98	8.03±4.14	11.22±8.62	8.81±6.48	14.16±4.98

Different letters in the same column indicate significant differences between groups according to the Tukey test. $^{a, b}$ Means in the same row with different superscript letters indicate differences ($p < 0.05$).

Table S4. Effects of the fruit extract of *K. coccinea* on immune organ index of white-feather broilers

items	CK	PD	LD	MD	HD
**0~21d**					
Liver	26.54±2.50	29.78±3.08	27.36±2.06	27.99±2.18	28.72±4.37
Thymus	3.23±0.76	3.47±1.15	3.68±0.81	3.69±0.85	3.73±0.99
Spleen	0.78±0.24^{b}	0.98±0.35^{ab}	1.01±0.24^{ab}	0.98±0.34^{ab}	1.18±0.44^{a}
Bursa	2.27±0.66^{ab}	1.66±0.29^{b}	1.92±0.53^{ab}	2.35±0.47^{a}	2.10±0.59^{ab}
**22~42d**					
Liver	20.53±2.63	18.96±1.83	19.57±2.60	19.81±2.16	19.16±3.36
Thymus	1.34±1.02^{b}	2.15±0.92^{ab}	1.97±0.77^{ab}	3.2±1.45^{a}	3.15±1.44^{a}
Spleen	1.19±0.44	1.45±0.47	1.38±0.26	1.45±0.35	1.29±0.21
Bursa	0.63±0.29	0.68±0.19	0.59±0.19	0.52±0.11	0.73±0.47

Different letters in the same column indicate significant differences between groups according to the Tukey test. $^{a, b}$ Means in the same row with different superscript letters indicate differences ($p < 0.05$).

Table S5. Effects of the fruit extract of *K. coccinea* on immunoglobulin levels in white-feather broilers

items	CK	PD	LD	MD	HD
**1~21d**					
IgG（μg/mL）	63.2±3.77^{a}	55.43±3.43^{b}	52.67±3.48^{b}	65.07±3.77^{a}	53.22±3.94^{b}
IgM（ng/mL）	2544.77±184.00^{c}	2679.65±169.18^{c}	3262.78±222.59^{b}	2602.57±306.82^{c}	3609.85±226.74^{a}
IgA（ng/mL）	7936.4±396.41^{b}	8807.09±490.27^{a}	6457.78±509.85^{c}	6573.52±523.53^{c}	8409.51±416.99^{ab}
C3（μg/mL）	804.87±37.56^{ab}	772.6±28.76^{bc}	843.87±4.37^{a}	671.12±38.04^{d}	749.08±38.39^{c}
C4（μg/mL）	431.24±20.01^{bc}	454.48±10.39^{b}	413.13±21.35^{c}	436.62±21.65^{bc}	492.77±28.21^{a}
**22~42d**					
IgG（μg/mL）	76.61±4.27^{a}	64.29±3.39^{b}	60.65±5.20^{b}	55.69±4.54^{c}	61.08±2.48^{b}
IgM（ng/mL）	4429.94±256.11^{a}	3151.75±250.51^{c}	3756.66±257.77^{b}	3756.89±186.94^{b}	3889.94±310.97^{b}
IgA（ng/mL）	9438.17±163.53^{a}	9762.24±224.80^{a}	7927.47±206.13^{b}	7910.41±492.66^{b}	8297.02±528.59^{b}
C3（μg/mL）	847.81±35.13^{b}	869.01±15.14^{b}	743.6±22.93^{d}	805.06±35.51^{c}	946.46±36.86^{a}
C4（μg/mL）	578.89±31.99^{a}	573.7±24.38^{a}	517.22±25.80^{b}	574.2±25.85^{a}	537.03±12.35^{b}

Different letters in the same column indicate significant differences between groups according to the Tukey test. $^{a, b}$ Means in the same row with different superscript letters indicate differences ($p < 0.05$).

Table S6. Effects of the fruit extract of *K. coccinea* on mRNA expression levels of cytokines in spleen of white-feather broilers

items	CK	PD	LD	MD	HD
**1~21d**					
IL-2	1.45±1.20^{a}	1.63±1.14^{a}	0.22±0.07^{c}	0.42±0.08^{b}	0.23±0.11^{c}
IFN-γ	1.52±1.35^{ab}	2.07±1.93^{a}	0.2±0.06^{b}	0.39±0.14^{b}	0.26±0.13^{b}
**22~42d**					
IL-2	1.18±0.78^{b}	1.56±0.67^{b}	0.82±0.5^{b}	3.78±3.4^{a}	1.93±1.05^{ab}
IFN-γ	1.18±0.79^{b}	1.77±0.81^{b}	0.88±0.54^{b}	4.37±4.24^{a}	1.59±0.82^{b}

Different letters in the same column indicate significant differences between groups according to the Tukey test. $^{a, b}$ Means in the same row with different superscript letters indicate differences ($p < 0.05$)

Table S7. Effects of the fruit extract of *K. coccinea* on antioxidant function of white-feather broilers

**items**	**CK**	**PD**	**LD**	**MD**	**HD**
**1~21d**					
T-AOC（U/mL）	0.67±0.03^{ab}	0.73±0.11^{ab}	0.74±0.12^{a}	0.74±0.11^{a}	0.61±0.12^{b}
SOD（U/mL）	85.29±5.38^{bc}	91.77±3.63^{a}	84.99±4.41^{c}	92.81±2.24^{a}	89.77±3.55^{ab}
MDA（nmol/mL）	23.46±4.0^{a}	21.53±4.32^{ab}	23.02±2.85^{a}	21.03±1.72^{ab}	19.36±1.02^{b}
GSH-Px（U/mL）	39.38±4.18^{b}	40.01±3.69^{ab}	35.80±1.69^{c}	43.03±2.22^{a}	39.19±1.53^{b}
**22~42d**					
T-AOC（U/mL）	0.88±0.07^{a}	0.73±0.11^{b}	0.88±0.07^{a}	0.87±0.05^{a}	0.85±0.06^{a}
SOD（U/mL）	76.97±6.76	80.05±11.82	75.33±7.80	82.74±4.24	76.37±8.42
MDA（nmol/mL）	30.73±1.82^{b}	29.74±2.84^{b}	28.54±1.86^{b}	31.68±2.42^{b}	36.64±5.80^{a}
GSH-Px（U/mL）	26.11±2.48^{c}	35.20±4.65^{a}	35.30±1.66^{a}	30.99±1.48^{b}	32.76±2.47^{ab}

Different letters in the same column indicate significant differences between groups according to the Tukey test. $^{a, b}$ Means in the same row with different superscript letters indicate differences ($p < 0.05$)

Table S8. Effects of the fruit extract of *K. coccinea* on intestinal villus length and crypt depth of white-feather broilers

**items**	**CK**	**PD**	**LD**	**MD**	**HD**
**1~21d**					
villus height	937.98±196.54	940.97±119.85	984.31±149.55	1094.80±262.90	1084.44±110.31
Crypt depth	217.11±29.46^{ab}	222.24±35.45^{ab}	236.41±39.32^{a}	180.72±33.15^{c}	194.72±13.42^{bc}
V/C	4.33±0.70^{b}	4.27±0.45^{b}	4.27±1.06^{b}	6.29±2.08^{a}	5.58±0.54^{ab}
**22~42d**					
villus height	1038.94±108.99^{c}	1199.22±189.57^{bc}	1173.51±200.78^{bc}	1335.82±175.96^{ab}	1429.80±171.76^{a}
Crypt depth	221.97±22.22	238.24±23.88	217.06±20.39	213.75±21.13	237.01±37.63
V/C	4.71±0.61^{c}	5.10±1.09^{bc}	5.44±1.01^{abc}	6.25±0.59^{a}	6.13±0.94^{ab}

Different letters in the same column indicate significant differences between groups according to the Tukey test. $^{a, b}$ Means in the same row with different superscript letters indicate differences ($p < 0.05$).
